# Motor Learning Deficits in a Neonatal Mouse Model of Hypoxic-Ischemic Injury

**DOI:** 10.3390/children12010027

**Published:** 2024-12-27

**Authors:** Maria Marlicz, Weronika Matysik, Emily Zucker, Sarah Lee, Hannah Mulhern, Jennifer Burnsed

**Affiliations:** 1 Department of Pediatrics, Division of Neonatology, University of Virginia, Charlottesville, VA 22908, USA; cwn4ex@virginia.edu (M.M.); wmm4q@virginia.edu (W.M.); hmm6cs@virginia.edu (H.M.); 2 School of Arts and Sciences, University of Virginia, Charlottesville, VA 22908, USA; eaz3tg@virginia.edu (E.Z.); hs4mj@virginia.edu (S.L.)

**Keywords:** hypoxic–ischemic encephalopathy, motor outcomes, cerebral palsy, neonate

## Abstract

Background/Objectives: Motor deficits following neonatal brain injury, from cerebral palsy to subtle deficits in motor planning, are common yet underreported. Rodent models of motor deficits in neonatal hypoxia–ischemia (HI) allow improved understanding of the underlying mechanisms and neuroprotective strategies. Our goal was to test motor performance and learning in a mouse model of neonatal HI. Methods: We induced HI in postnatal day (p)10 C57/Bl6 mice through unilateral carotid ligation followed by 60 min of 8% oxygen exposure, or a sham procedure. At p30, we assessed complex motor performance and learning using the accelerating rotarod and complex running wheel tasks. Results: In the rotarod task, HI mice performed worse than sham mice, with shorter latencies to fall (n = 6 sham, 9 HI; day 1, *p* = 0.033; day 2, *p* = 0.013; day 3, *p* = 0.023). Sham mice demonstrated improved performance across days (*p* = 0.005), and HI mice did not (*p* = 0.44). During the simple running wheel task, we observed no difference in wheel rotation and speed between groups (n = 5/group; day 1, *p* = 0.67; day 4, *p* = 0.53). However, when navigating a wheel with a random pattern of spokes removed (complex task), HI mice took longer than sham mice to reach a plateau in performance (n = 5/group; day 1, *p* = 0.02; day 4, *p* = 0.77). Conclusions: Our findings demonstrate that young adult mice exposed to HI exhibit significant deficits and delayed learning in complex motor performance compared to sham mice. HI mice do not show deficits in gross motor performance; however, more subtle impairments are present in complex motor performance and learning. This HI model exhibits subtle motor deficits relevant to findings in humans and may be a useful tool in testing further neuroprotective strategies.

## 1. Introduction

Motor deficits following neonatal brain injury are common, with a broad spectrum of outcomes, ranging from cerebral palsy (CP) to deficits in motor planning, learning, and coordination. While cerebral palsy occurs in about 15–20% of children after neonatal hypoxic–ischemic encephalopathy (HI), other motor deficits, such as problems with motor coordination, motor control, and processing speed, are very common, occurring in over half of those without a CP diagnosis [1,2]. These other neuromotor deficits are likely underreported, as they are more subtle, may not present until preschool or school age, and are often not routinely assessed in at-risk children. However, motor deficits have a significant impact on children’s lives, affecting academic achievement and daily activities, such as participation in sports [3].

Neonatal hypoxia–ischemia is the most common cause of brain injury in the full-term neonate and a common cause of neurodevelopmental deficits. The Vannucci model, a unilateral permanent carotid ligation followed by global hypoxia, has been the most commonly used rodent model of neonatal HI for the past 40 years [4,5,6]. While this model does not result in a cerebral palsy phenotype [7], more subtle differences in motor skills and motor learning in this model have not been widely studied. Further study of motor learning and planning in this widely used model is important, as more subtle motor deficits are common following neonatal HI.

The goal of this study was to test whether this commonly used neonatal HI mouse model exhibits subtle deficits in motor planning, learning, and coordination using a battery of behavioral tasks.

## 2. Materials and Methods

### 2.1. Animals

All mice used in this study were handled according to the University of Virginia Animal Care and Use Committee-approved protocol. Animal housing was in accordance with the National Institutes of Health Guide for the Care and Use of Laboratory Animals [8]. Our breeding colony of C57Bl/6 mice produced all mice used in this study. Mice were used from across different breeders to help control for litter effects. Weaning was between postnatal day (p)20 and 22, per our ACUC protocols. Housing and enrichment were in accordance with our standard ACUC protocols. Mice from both groups (HI and sham) were housed individually during the running wheel experiments to allow them uninterrupted access to the running wheels. Mice of both sexes were used in all experiments.

### 2.2. Hypoxia–Ischemia or Sham Procedure

HI injury was created in p10 mice as previously described (permanent unilateral carotid ligation followed by 60 min of hypoxia at FiO_2_ = 0.08) [4,6]. The sham procedure involved a neck incision and equivalent anesthesia exposure without carotid ligation or hypoxia exposure. Normothermia was maintained using circulating water warming mats under the surgical field and hypoxia chamber. Body temperature checks were performed throughout the experiment using an infrared detector. Ambient temperature was continuously monitored in the hypoxia chamber. Roughly one hour following ligation, mice in the HI group were exposed to hypoxia. Mice were then returned to the dam and allowed to grow normally until undergoing behavioral testing.

### 2.3. Rotarod Experiment

Seven days prior to the start of the behavioral task, mice were habituated to handling (daily for 10–15 min by a consistent researcher) to reduce stress during behavioral testing. An accelerating rotarod was used for these experiments (ENV-574M Five Lane Rota-Rod, Med Associates, Latham, NY, USA). The apparatus consisted of five striated cylinders placed 20 cm above the floor, each separated by a plastic wall. In order to habituate to the experimental room, mice were placed in the room an hour before the experiment each day.

First, animals were placed on the rod with constant low-speed rotation to become used to the activity. During each trial, a single, consistent researcher placed the mouse on the stationary rotarod. Once placed, the rotarod was set to accelerate from 4 to 40 rpm gradually during a 300 s interval. The time to fall (latency) was measured for each mouse using an infrared beam and automated detection software that recorded the time when the beam was broken by the mouse’s fall. If the mouse did not fall from the rod during the 300 s trial, it was removed and the latency time was recorded as 300 s.

**Figure 1:**
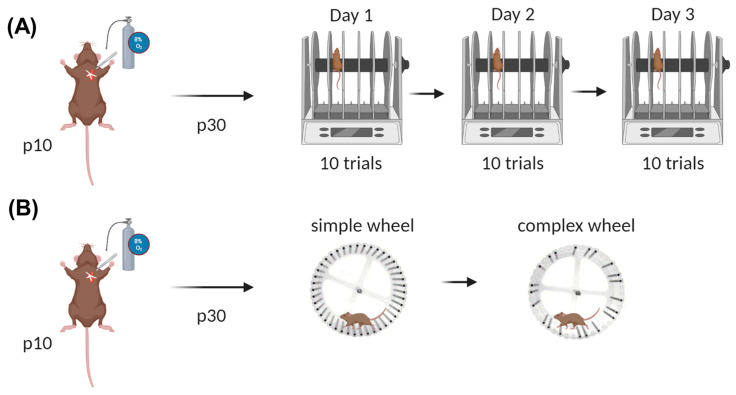
Experimental timeline. (**A**) Rotarod experiment. (**B**) Running wheel experiment.

Ten trials were performed daily for three days (Figure 1A) based on prior studies that used this paradigm as a test of both short-term (across trials/within day) and long-term (across days) motor learning [9]. The time between each trial was 300 s. Latency to fall was analyzed across trials daily (to examine short-term learning) and across days (to examine long-term learning). Researchers performing all behavioral experiments were blinded to the experimental group. The rotarod trials were performed consistently between 1 and 5 PM.

### 2.4. Running Wheel Experiment

This running wheel task uses a motor skill sequence (MOSS) to test gross motor skills, locomotion, and motor learning [10,11,12,13,14,15]. On p30, mice were placed in an individually housed cage with open access to a simple running wheel for a week (Figure 1B). Using LabChart Pro software (version 8), the number of revolutions of the wheel, time per revolution, and timestamps for each revolution were recorded. After 4 days, the wheel was modified to remove a random pattern (consistent design across mice) of spokes, which the mice then had to learn to navigate. This is referred to as the “complex wheel” (Figure 1B). Mice were then placed back in the cage with open access to the complex wheel, and the same metrics listed above were recorded. As in the rotarod experiments, mice were handled for habituation for the week prior to starting the task. On p29, each mouse had its whiskers trimmed in order to prevent it from using them to detect the upcoming wheel rungs during navigation of the wheel [11].

The recorded number of wheel revolutions and time per revolution were extracted from LabChart (version 8) and imported into GraphPad Prism (version 10.4.1). Any revolutions that took longer than two seconds were excluded from analysis in order to account for instances when mice were sitting dormant on the wheel and not actively running. Revolutions were binned by time of day and analyzed.

### 2.5. Data Analysis

GraphPad Prism was used for all data analysis, including descriptive statistics and further data analysis. For the rotarod behavior test, we initially conducted a two-way repeated measures (RM) ANOVA for day, trial, and interaction. None were significant, so the mean latency to fall across the 10 trials within each day was used in a two-way RM ANOVA for day and treatment group. In the running wheel task, we used an unpaired t-test to compare performance between the two groups each day. For each finding, we calculated effect sizes using Cohen’s d, which is reported below. Statistical significance was set as a *p*-value < 0.05. Mean and standard deviation are reported unless otherwise noted below.

## 3. Results

### 3.1. Animal Groups

A total of 23 C57Bl/6 mice were used in the rotarod experiments. Five mice died during hypoxia–ischemia (21%). Of the 18 that survived, 9 mice (5 females and 4 males) were in the HI group and 6 were in the sham procedure group (5 females and 1 male). Three mice in total were excluded (one sham and two HI females) due to their unwillingness to run or because they stopped or jumped off the rod.

A total of 13 C57Bl/6 mice were used in the running wheel experiments. Three mice died during HI (23%). Of the 10 that survived, 5 were in the HI group (3 females and 2 males) and 5 were in the sham group (2 females and 3 males). None were excluded.

### 3.2. Young Adult Mice Exposed to Neonatal HI Exhibit Impaired Learning During Rotarod Task

**Figure 2:**
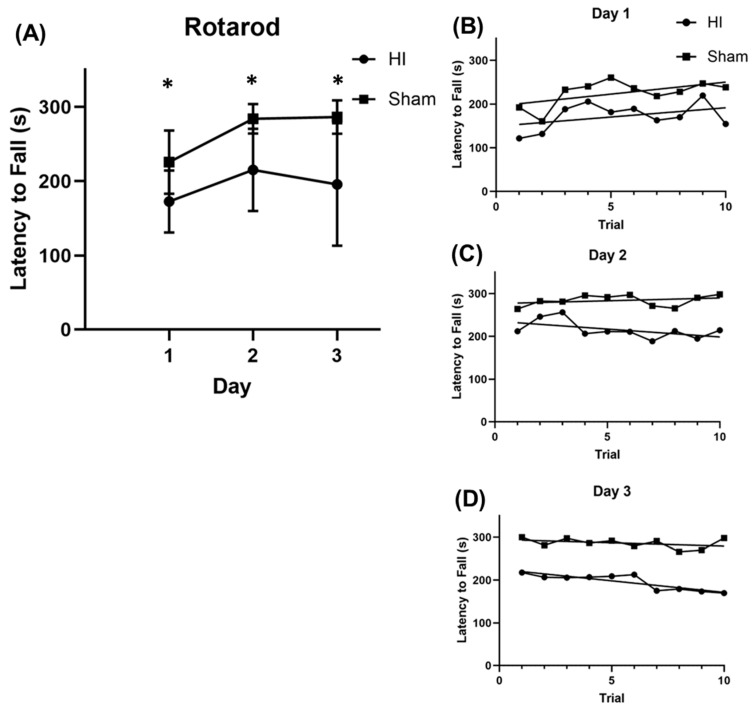
HI mice exhibit worse performance and decreased learning across days on a rotarod learning task. (**A**) Mean latency to fall on days 1–3 in HI and sham groups (day 1, HI: 172.5+/−41.68 s vs. sham: 225.5+/−42.66 s, *p* = 0.033; day 2, HI: 215.2+/−55.41 s vs. sham: 283.9+/−19.87 s, *p* = 0.013; day 3, HI: 195.7+/−82.67 s vs. sham: 286.1+/−22.59 s, *p* = 0.023, n = 9 HI, n = 6 sham). (**B**–**D**) Mean latency to fall in HI and sham groups across 10 trials on days 1–3 ((**B**–**D**), respectively). (There were no significant differences in either group between trial performance within days (sham, *p* = 0.076; HI, *p* = 0.36 for trial, RM two-way ANOVA).). * *p* < 0.05.

Mice in the HI group exhibited worse performance on a rotarod learning task across all three training days compared to sham mice. On days 1–3, HI mice had a shorter mean latency to fall (averaged across 10 trials per day per mouse) compared to sham mice (Figure 2A, day 1, HI: 172.5+/−41.68 s vs. sham: 225.5+/−42.66 s, *p* = 0.033; day 2, HI: 215.2+/−55.41 s vs. sham: 283.9+/−19.87 s, *p* = 0.013; day 3, HI: 195.7+/−82.67 s vs. sham: 286.1+/−22.59 s, *p* = 0.023; n = 9 HI, n = 6 sham). This finding was confirmed when we performed a repeated measures two-way ANOVA, which showed that day and treatment group were both significant (HI vs. sham, *p* = 0.01; day, *p* = 0.002), but the interaction of the two was not (*p* = 0.27). The effect size for each day was 1.26 on day 1, 1.65 on day 2, and 1.49 on day 3 (Cohen’s d). There were no significant differences in either group between trial performance within days (sham, *p* = 0.076; HI, *p* = 0.36 for trial, RM two-way ANOVA) (Figure 2D).

### 3.3. After Neonatal HI, Young Adult Mice Exhibit Impaired Motor Learning During the Complex Wheel but Do Not Exhibit Gross Motor Deficits

**Figure 3:**
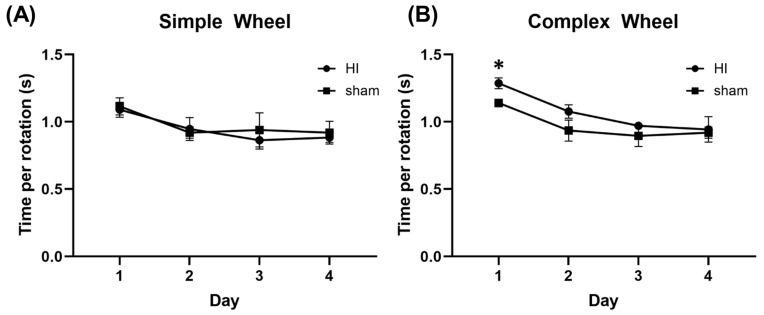
HI mice perform similarly to sham mice on the simple running wheel but are slower to navigate the complex running wheel. (**A**) Performance on the simple wheel was similar between HI and sham groups (day 1 mean speed, HI group: 1.09 ± 0.05 s/rotation vs. sham: 1.12 ± 0.07 s/rotation, *p* = 0.67; day 4 mean speed, HI group: 0.88 ± 0.04 s/rotation vs. sham: 0.92 ± 0.05 s/rotation, *p* = 0.53; n = 5 mice/group). (**B**) HI mice were initially slower to navigate the complex wheel (day 1, HI: 1.29 ± 0.04 s/rotation vs. sham: 1.14 ± 0.02 s/rotation, *p* = 0.02; day 2, HI: 1.08 ± 0.05 s/rotation vs. sham: 0.94 ± 0.02 s/rotation, *p* = 0.08; day 3, HI: 0.97 ± 0.04 s/rotation vs. sham: 0.895 ± 0.04 s/rotation, *p* = 0.18; day 4, HI: 0.94 ± 0.07 s/rotation vs. sham: 0.92 ± 0.07 s/rotation, *p* = 0.77, * *p* < 0.05).

During the first phase of the task, mice were placed in individual housing with open access to run on a simple running wheel. There was no difference in the speed at which the HI or sham groups turned the wheel, as measured by the number of seconds it took to rotate the wheel (Figure 3A, n = 5 mice/group; day 1 mean speed, HI group: 1.09 ± 0.05 s/rotation vs. sham: 1.12 ± 0.07 s/rotation, *p* = 0.67; day 4 mean speed, HI group: 0.88 ± 0.04 s/rotation vs. sham: 0.92 ± 0.05 s/rotation, *p* = 0.53). The effect size for the simple wheel on day 1 was 0.49, and on day 4 it was 0.88 (Cohen’s d).

Following open access to the simple wheel, mice were briefly removed from the cage while the standard pattern of rungs was removed to create the complex wheel. Mice were then placed back in the cage with open access to the complex wheel. On the first day of complex wheel navigation, HI mice took significantly longer per rotation of the complex wheel than they had the prior day on simple wheel (Figure 3B, HI simple day 4: 0.88 ± 0.04 s/rotation vs. HI complex day 1: 1.29 ± 0.04 s/rotation, *p* = 0.0002; sham simple day 4: 0.92 ± 0.05 s/rotation vs. sham complex day 1: 1.14 ± 0.02 s/rotation, *p* = 0.074). The sham group was significantly faster at navigating the complex wheel compared to the HI group on day 1 (HI: 1.29 ± 0.04 s/rotation vs. sham: 1.14 ± 0.02 s/rotation, *p* = 0.02). However, by day 2 HI mice began to catch up to the performance of sham mice (HI: 1.08 ± 0.05 s/rotation vs. sham: 0.94 ± 0.02 s/rotation, *p* = 0.08). Similarly, on days 3 and 4, there was no significant difference in speed between the HI and sham groups (HI day 3: 0.97 ± 0.04 s/rotation vs. sham day 3: 0.895 ± 0.04 s/rotation, *p* = 0.18; HI day 4: 0.94 ± 0.07 s/rotation vs. sham day 4: 0.92 ± 0.07 s/rotation, *p* = 0.77). The effect size for the complex wheel was 4.74 on day 1, 3.67 on day 2, and 1.85 on day 3 (Cohen’s d).

## 4. Discussion

The goal of this study was to compare motor learning in young adult mice exposed to neonatal HI with those subjected to the sham procedure. We found that mice exposed to neonatal HI exhibited deficits in motor learning in two separate tasks performed in young adulthood. Consistent with prior studies, the HI group did not have any gross motor deficits at baseline when compared to sham mice. However, they did not show improvement in performance over several days during a multi-day rotarod learning task and were slower to learn to navigate a complex running wheel, indicating a motor learning deficit.

Prior studies have not found that this mouse model of HI exhibits a cerebral palsy phenotype or gross motor deficits. This is consistent with our finding that there were no differences in performance on the simple running wheel. However, a few studies have found minor motor abnormalities of various types in the model [16,17]. Clinically, cerebral palsy is less common in human survivors of neonatal HI compared to deficits in motor coordination, fine motor control, and motor learning [18]. A prior study found that rodents exhibited deficits in ladder rung walking after exposure to neonatal HI [16] but did not find differences between HI and uninjured mice in a two-day rotarod task. However, that study did not examine differences in performance over time that would indicate long-term motor learning, as we did in this study. Similar to our findings, Cengiz et al. [17] found that mice exposed to HI showed impaired motor learning on a similar rotarod task over three days.

Some deficits in learning have been demonstrated in this model before. Adult mice exposed to neonatal HI have been shown to have deficits in spatial learning and memory using a Morris water maze task [17], as well as impaired learning during more sophisticated tasks such as a touchscreen assessment [19]. Our findings in the complex wheel task demonstrate that on the first day, the HI mice were slower to navigate the wheel; however, they eventually caught up to the performance of the sham mice. This indicates that motor learning is impaired but that, with training, the HI mice are able to catch up. Our prior work demonstrated that there is increased abnormal neuronal activity in the learning and motor centers, such as the hippocampus, motor cortex, and thalamus, during neonatal HI seizures in this model [20]. Early, abnormal neuronal activation in these centers may lead to changes in synaptic and circuit development, leading to deficits in motor learning. Future studies will examine how these changes lead to the motor learning deficits observed in this model.

The limitations of this study include the well-described variability in this widely used neonatal hypoxia–ischemia model [5,21,22,23]. The two behavioral assays used have been employed in several studies as tests of motor learning [9,11]; however, motor learning is complex, and performance may also be impacted by the rodent’s fine motor control, coordination, and motivation. The sample size was limited, and further studies should be powered to examine sex differences in motor learning after neonatal HI. As part of the protocol for the complex running wheel, mice had their whiskers trimmed in order to prevent them from detecting rungs during performance of the task [11], which required additional handling and potential stress to the rodents. Although all mice across groups were exposed to this, it may have impacted results.

These results showing motor learning deficits in the most widely used rodent model of neonatal hypoxic–ischemic encephalopathy will allow further use of this model in examining the effect of early-life injury on motor and learning circuits and in testing the effect of therapies on motor learning in this disease. Over half of human infants exposed to neonatal HI have motor deficits other than cerebral palsy, such as deficits in motor coordination, motor control, and motor learning [1,18], making this aspect of the model an important and useful tool for preclinical studies in neonatal HI.

## 5. Conclusions

This widely used rodent model of neonatal HI exhibits motor learning deficits that will be useful in further preclinical studies of this disease.

## Data Availability

The original contributions presented in this study are included in the article. Further inquiries can be directed to the corresponding author.
